# Neurologic and Neuroscientific Evidence in Aged COVID-19 Patients

**DOI:** 10.3389/fnagi.2021.648662

**Published:** 2021-03-23

**Authors:** Shraddha Mainali, Marin E. Darsie

**Affiliations:** ^1^Department of Neurology, The Ohio State University, Columbus, OH, United States; ^2^Department of Emergency Medicine, University of Wisconsin Hospitals and Clinics, Madison, WI, United States; ^3^Department of Neurological Surgery, University of Wisconsin Hospitals and Clinics, Madison, WI, United States

**Keywords:** COVID-19, SARS-CoV-2, neurologic complications, elderly, aged adults, older patients, neurologic manifestations, neuro

## Abstract

The COVID-19 pandemic continues to prevail as a catastrophic wave infecting over 111 million people globally, claiming 2. 4 million lives to date. Aged individuals are particularly vulnerable to this disease due to their fraility, immune dysfunction, and higher rates of medical comorbidities, among other causes. Apart from the primary respiratory illness, this virus is known to cause multi-organ dysfunction including renal, cardiac, and neurologic injuries, particularly in the critically-ill cohorts. Elderly patients 65 years of age or older are known to have more severe systemic disease and higher rates of neurologic complications. Morbidity and mortality is very high in the elderly population with 6–930 times higher likelihood of death compared to younger cohorts, with the highest risk in elderly patients ≥85 years and especially those with medical comorbidities such as hypertension, diabetes, heart disease, and underlying respiratory illness. Commonly reported neurologic dysfunctions of COVID-19 include headache, fatigue, dizziness, and confusion. Elderly patients may manifest atypical presentations like fall or postural instability. Other important neurologic dysfunctions in the elderly include cerebrovascular diseases, cognitive impairment, and neuropsychiatric illnesses. Elderly patients with preexisting neurologic diseases are susceptibility to severe COVID-19 infection and higher rates of mortality. Treatment of neurologic dysfunction of COVID-19 is based on existing practice standards of specific neurologic condition in conjunction with systemic treatment of the viral illness. The physical, emotional, psychologic, and financial implications of COVID-19 pandemic have been severe. Long-term data are still needed to understand the lasting effects of this devastating pandemic.

## Introduction

Over the course of one year, the novel SARS-CoV-2 virus has wreaked havoc globally accounting for over 111 million documented infections with a vast toll of death and disability. Although the proportion of severe cases is likely to depend on the study population and epidemiological behavior of the infection in each country or geographic region, current evidence suggests that older individuals and those with compromised immune systems are more likely to develop severe forms of COVID-19. In a large Chinese case series of 72,314 cases, older adults were more susceptible to the virus with case fatality rate of 8.0% in patients aged 70–79 years and 14.8% in patients aged ≥80 years (Wu and McGoogan, 2020). Some studies have reported age >65 years as an independent predictor of mortality (Wu et al., 2020). Countries like Italy that have endured severe effects of the pandemic have also experienced highest morbidity and mortality in the elderly population, particularly those with underlying comorbidities (Livingston and Bucher, 2020). In fact, Italy has one of the world's oldest population, with 23% of people ≥65 years, which is likely the reason for Italy's high case fatality rate (7.2%) compared to other countries (Onder et al., 2020). In the United States (U.S.), although infection is most prevalent in the 18–29 age group (23%), elderly patients ≥65 years are 5–13 times more likely to be hospitalized and have 9–630 times higher likelihood of death; with the highest rate of hospitalization and death among patients ≥85 years of age (Centers for Disease Control Prevention, 2020). Per the National Vital Statistics System data on the demographic and geographic trends of COVID-19 in the U.S between May 1 and August 31, 78.2% of deaths occurred predominantly in men (53.3%) aged ≥65 years (Gold et al., 2020). Neurologic dysfunctions of COVID-19 are also more common in the elderly population (Frontera et al., 2020), and the presence of neurologic dysfunction has been identified as an independent predictor of mortality in hospitalized COVID-19 patients (Aggarwal et al., 2020; Frontera et al., 2020; Pranata et al., 2020).

As neurologic complications are often associated with disease severity and mortality, characterization of neurologic dysfunction and accurate prognostication is crucial. Here we briefly discuss common neurologic dysfunctions of COVID-19 in the elderly, possible determinants of disease severity in this age group, outcomes, and treatment.

## Neurologic Associations and Complications in Aged COVID-19 Patients

The most commonly reported symptoms of COVID-19 are fever, dry cough, fatigue, and dyspnea (Siordia, 2020). Elderly patients are likely to present with atypical symptoms such as falls, postural instability, or delirium (Steinmeyer et al., 2020). The pulmonary manifestations of COVID-19 drive most hospitalizations worldwide, more commonly in patients ≥65 years and those with comorbidities like diabetes and hypertension (Xu et al., 2020). Critically-ill COVID-19 patients frequently develop multi-organ dysfunction including arrhythmias, myocardial injury, heart failure, arterial and venous thrombosis, disseminated intravascular coagulation (DIC), dysregulated immune response, and neurologic complications (DeKosky et al., 2020; Guzik et al., 2020; Ortiz-Prado et al., 2020). Preexisting vascular disease is known to predispose individuals to severe COVID-19 infection, with aged individuals being more susceptible to more severe courses (Guzik et al., 2020; Li et al., 2020).

Neurologic complications are noted to be more common in patients with severe respiratory illness (Mao et al., 2020). Furthermore, neurologic dysfunctions can also lead to longer duration of mechanical ventilation in critically-ill patients (Helms et al., 2020). Neurologic dysfunctions of COVID-19 span a spectrum of mild symptoms like headache, anosmia, ageusia to severe complications including stroke, meningitis, encephalitis, and status epilepticus (DeKosky et al., 2020; Iadecola et al., 2020; Mao et al., 2020; Paterson et al., 2020). Heterogeneity in reports related to data elements, population, study design, analysis, and associated comorbidities have precluded a robust systematic analysis of neurologic dysfunctions of COVID-19. A qualitative, systematic review on clinical features of COVID-19 specifically in the elderly cohort has reported several neurologic manifestations including headache, fatigue, myalgias, dizziness, gait disturbances, confusion, and delirium (Neumann-Podczaska et al., 2020). The severity of COVID-19 illness and associated neurologic complications are known to be more prevalent in persons with preexisting comorbidities such as cerebrovascular and cardiovascular diseases, hypertension, diabetes, obesity, respiratory diseases, and malignancy (Worldometer, 2020; Zádori et al., 2020).

Acute encephalopathy has been reported as a common neurologic complication of COVID-19, particularly in elderly patients and those with severe diseases (Garg et al., 2020; Helms et al., 2020). Across the literature, various terms have been used to describe altered cognition including acute encephalopathy, delirium, altered mental status, acute confusional state, acute brain dysfunction, acute brain failure, etc. Lack of standard nomenclature with varied reporting practices related to conceptual or semantic disparities across different medical disciplines has made it difficult to pool data for evaluating the true global prevalence and impact of encephalopathy related to COVID-19. Although a consensus statement of 10 critical care societies was published in early 2020 proposing the standard nomenclature (Slooter et al., 2020), existing literature on COVID-19-related-encephalopathy remain tainted with a variety of terms and reporting practices. Historically, delirium has been noted in about 10–15% of elderly patients presenting to the hospital and diagnosed in up to one-third of general medical patients ≥70 years (Marcantonio, 2017). However, it is much more common in critically-ill patients. Although reports are variable across studies, one multicenter study reported a delirium prevalence of up to 73% in intubated patients with ARDS (Hsieh et al., 2015). In comparison, published reports have suggested increased prevalence of delirium in COVID-19 patients presenting to the emergency department with estimates of around 28% and much higher rates in critical care cohorts of up to 84.3% (Helms et al., 2020; Kennedy et al., 2020; Liotta et al., 2020). In particular, elderly patients with comorbidities are particularly vulnerable, with delirium at presentation noted in up to 36.8% of elderly patients (Poloni et al., 2020). This apparent increase in the prevalence of delirium during the COVID-19 pandemic is likely to be due to both infectious as well as environmental factors such as social isolation (LaHue et al., 2020). Moreover, while acute encephalopathy is more common in older COVID-19 patients with medical comorbidities, when controlled for age and comorbidities, the presence of acute encephalopathy was still noted to be a predictor of critical care need, intubation, and 30-day mortality (Shah et al., 2020).

Acute stroke is another commonly reported neurologic complication of COVID-19, particularly in the elderly population (Berardelli et al., 2020; Josephson and Kamel, 2020). Li et al. reported a 5% risk of ischemic stroke and a 0.5% risk of cerebral hemorrhage in 221 patients with COVID-19 infection from Wuhan, with the highest prevalence in aged individuals with underlying vascular and thrombotic risk factors such as hypertension, diabetes, and elevated plasma D-dimer levels (Li et al., 2020). Recent systematic reviews report stroke incidence of 1–2% in COVID-19 patients, which is significantly higher than historical controls, including a cohort with seasonal influenza (Fridman et al., 2020; Katsanos et al., 2020; Merkler et al., 2020; Nannoni et al., 2020). Mortality among hospitalized COVID-19 patients with acute stroke is also extremely high. Reports have noted mortality rates of 31.5–34.4%, with highest impact in older patients (Fridman et al., 2020; Nannoni et al., 2020). Elderly stroke survivors are also at increased risk of severe infection and suffer from higher mortality, likely related to their underlying comorbidities and swallowing complications (Aggarwal et al., 2020; Pranata et al., 2020). A recent meta-analysis of 39 studies on COVID-19-associated stroke suggested a mean age of 63.4 ± 13.1 years with male predominance and clinical findings of elevated D-dimer, elevated fibrinogen, and the presence of antiphospholipid antibodies (Tan et al., 2020). Although many other neurologic and neuropsychiatric symptoms have been reported, focused reports on elderly is generally sparse.

## Aging Process, COVID-19 Disease Severity and Neurologic Impairment: Social and Pathophysiological Determinants

Multiple postulations have been made regarding mechanisms of disease severity in the elderly, including higher rates of neurologic complications of COVID-19 (Banerjee and Viswanath, 2020; Chen et al., 2020; Connors and Levy, 2020; Mehta et al., 2020; Paniz-Mondolfi et al., 2020; Sardu et al., 2020; Zhang et al., 2020). Investigators have recently performed integrative analyses of single-cell atlases in the lung and airways and across tissues to identify cell types and tissues that have the key molecular machinery required for SARS-CoV-2 infection (Muus et al., 2020). They further examined the relationship between specific cell types and three key covariates of disease severity including age, sex, and smoking status. The investigators noted increasing trends of double-positive ACE2^+^TMPRSS2^+^ cell proportions with *increasing age* which could be one of the reasons for disease severity in the elderly. Investigators also noted the presence of these receptors in tissues beyond the respiratory system, including oligodendrocytes in the brain. Furthermore, myelin proteins were noted to be co-expressed in numerous ACE2^+^TMPRSS2^+^ cells across various organs, which may suggest the potential for neurologic autoimmunity through the invasion of ACE2^+^TMPRSS2^+^ cells in organs such as the lungs and gut.

Frailty is common among the elderly (Bhaskar et al., 2020). It is characterized by declining function across several homeostatic systems leading to increased vulnerability to stressors and increased risk of adverse health outcomes (Maltese et al., 2020). Studies have shown that progressive physical frailty in the elderly is strongly correlated with declining cognition, which may in part be due to a common pathologic basis (Buchman et al., 2014; Wallace et al., 2019). Elderly patients with macroinfarcts, Alzheimer's disease, and Parkinson's disease pathology show independent association with the rate of change of both physical frailty and cognition (Buchman et al., 2014), placing these individuals at higher risk of COVID-19 disease severity.

The presence of chronic neurologic diseases (CND) has been reported to be an independent predictor of COVID-19 related death when controlled for confounding variables including, age, sex, baseline function, hypertension, diabetes, smoking, cardiac disease, pulmonary disease, and malignancy (García-Azorín et al., 2020). In a Spanish study, patients with CND were noted to be older with higher baseline disability, had more vascular risk factors, and exhibited fewer typical clinical symptoms of COVID-19, such as cough or malaise. Of the CND, elderly patients more commonly have preexisting neurovascular and neurodegenerative conditions, which in turn predisposes them to COVID-19 infection. Patients with neurodegenerative conditions like Parkinson's disease are vulnerable to severe COVID-19 illness due to older age, bulbar symptoms, respiratory dysfunction, frailty, and cognitive impairment. Similarly, patients with Alzheimer's disease and related dementias are at increased COVID-19 infection risk with a higher likelihood of morbidity and mortality (Brown et al., 2020).

Older patients are particularly vulnerable to the psychological burden of COVID-19. A recent report of a cross-sectional survey from Greece involving participants over the age of 60 years reported high rates of disrupted sleep (37.9%), moderate to severe depression (81.6%), and moderate to severe anxiety (84.5%) (Parlapani et al., 2020). Loneliness related to quarantine and social isolation has significantly impacted mental health outcomes in the elderly (Okruszek et al., 2020). Patients with CND are particularly vulnerable to social isolation. A recent nation-wide multi-center caregiver survey evaluated acute psychological effects of quarantine in frail elderly subjects with diagnoses of Alzheimer disease, dementia with Lewy bodies, frontotemporal dementia, and vascular dementia (Cagnin et al., 2020). This study including survey from 4,913 caregivers showed that quarantine induced a rapid increase of behavioral and psychological symptoms in ~60% of patients and stress-related symptoms in two-thirds of caregivers. Most common symptoms included irritability, apathy, agitation anxiety, and sleep disorder (Cagnin et al., 2020). Similarly, a Spanish study using online survey of PD patients showed that 67.5% patients perceived worsening symptoms during the quarantine period (Santos-García et al., 2020).

Furthermore, older adults have aging immune systems characterized by two main processes including immunosenescence and inflammaging (Mueller et al., 2020; Thomas et al., 2020). Immunosenescence is a process of gradual decline in innate (ineffective pathogen recognition or macrophage activation and reduction in natural killer cell cytotoxicity) and adaptive (thymic hypoactivity and reduction in the accumulation of anergic memory lymphocytes) immune functions leading to impairment in pathogen recognition, alert signaling, and clearance. Inflammaging is characterized by a hyperactive yet ineffective alert system with age-related process of systemic inflammation and autoimmune predisposition (Thomas et al., 2020). Emerging literature suggests that chronic systemic comorbidities and aging (known risk factors for severe COVID-19 disease) are associated with upregulation of inflammation and predisposition to neuroinflammatory and neurodegenerative conditions (Bossù et al., 2020; Scheiblich et al., 2020). Neuroinflammation also results from sustained anti-viral immune response with propagation of inflammation from the periphery to the brain through various pathways as previously described (Dantzer et al., 2008; Capuron and Miller, 2011). Moreover, neuroinflammation may alter integrity of the blood brain barrier, further enabling entry of circulating immune cells to the brain resulting in various neuropathological processes such as anxiety, depression, fatigue, psychomotor slowing, anorexia, cognitive dysfunction, and sleep impairment (Capuron and Miller, 2011), all of which are commonly reported with COVID-19.

Another hypothesis regarding T-cell paucity is related to exhaustion of immune system driven by repeated exposures to viruses over one's lifespan. This has been noted in patients with cytomegalovirus (CMV) seropositivity where cycles of reemergence is associated with immune remodeling and exhaustion of CD8^+^-T-cells leading to higher mortality in the elderly population (Olsson et al., 2000; Pawelec, 2014). Some other studies have indicated that T-cell depletion in elderly is likely related to cumulative exposure to various pathogens over the years (Ongrádi and Kövesdi, 2010; Bartlett et al., 2012). At the chromosomal level, the age-related immunomodulation is likely due to telomere shortening in viral-specific memory CD8^+^-T-cells, leading to a state of cell cycle arrest and hyper-inflammation, thereby limiting the ability to mount appropriate immune response against novel viral infections such as SARS-CoV-2 (Bellon and Nicot, 2017). Due to the neuroinflammatory state, patients with CND may be particularly challenged in this aspect. There are other reports of vascular inflammation with increased oxidative stress and endothelial dysfunction leading to microvascular disease which may play a role in increased incidence of COVID-19-related cerebrovascular diseases in the elderly (Ungvari et al., 2018). Studies have also reported that aging expands clonal populations of CD8^+^-T-cells restricting their diversity, while CD4^+^-T-cells retain the T-cell receptor reserve but suffer from activation deficits (Salam et al., 2013; Yoshida et al., 2017). A recent study on supercentenarians (>110 years) using single-cell transcriptome analysis of 61,202 peripheral blood mononuclear cells (PBMCs), showed that supercentenarians have an unusual population of cytotoxic CD4^+^-T-cells whose activation doesn't decline with age and can take on the effector functions normally performed by CD8^+^-T-cells (Hashimoto et al., 2019). Preservation of immunity through this mechanism might explain why some very old individuals with COVID-19 infection survive the pandemic.

Multiple other postulations regarding COVID-19 disease severity and increased mortality in the elderly have been proposed including age-related epigenetic changes, inflammasome activity, covalent modifications of human, and viral proteins etc. (Mueller et al., 2020). Further research is needed to understand the key factors leading to the vulnerability of the elderly population especially in regards to its intersection with aging and neuroscience.

## Treatment and Treatment Response of Aged COVID-19 Patients

As we approach one year since the World Health Organization's (WHO) initial declaration of the novel coronavirus pandemic, limited evidence-based therapies specific to COVID-19 are available with management primarily focused on treatment of associated complications and supportive care. As of early December 2020, the only antiviral agent to receive FDA approval for the treatment of COVID-19 is remdesivir [National Institute of Health (NIH), 2020]. The primary benefits are shortened time to recovery and reduction in the progression of disease in individuals at high risk of hyper-inflammation, who are diagnosed early (≤10 days of illness) and require supplemental oxygen (Young et al., 2021). The WHO and National Institutes of Health also recommend the use of dexamethasone, after the RECOVERY Trial demonstrated improved survival in hospitalized patients requiring supplemental oxygen [National Institute of Health (NIH), 2020]. Regarding management of neurologic complications of COVID-19, existing evidence-based therapies are used for specific neurologic conditions, in conjunction with systemic treatment with antivirals, corticosteroids, and immunomodulators, as appropriate.

Delirium management has long been a priority in the care of older adults. Multidisciplinary care focused on prevention of delirium using non-pharmacologic strategies, is the best practice (Oh et al., 2017). Non-pharmacologic interventions include patient-centered care with frequent re-orientation, regular visits from family and friends, optimization of hearing and vision by ensuring access to hearing aids and glasses, adequate hydration, adequate sleep, early mobilization, and minimization of unnecessary lines, tubes, polypharmacy, and precipitating medications (Oh et al., 2017; Kotfis et al., 2020). In centers with pandemic-related visitation restrictions, care teams can help meet the emotional needs by displaying family photos, facilitating the use of technology to connect patients with their families, and assessing patients' desire to access spiritual care. Early mobilization is the single intervention with the strongest evidence of decreasing the incidence and the length of delirium (Marra et al., 2017). Pre-COVID literature suggests that delirium can be prevented with appropriate in-hospital multimodal approach in about 30% of cases (Siddiqi et al., 2016). Regular clinical assessments for pain, agitation, and delirium with validated screening tools can help with early recognition and management (Oh et al., 2017; Kotfis et al., 2020). As delirium/acute encephalopathy is a common manifestation of COVID-19, it is important to establish a baseline mental status for all hospitalized COVID-19 patients (Oh et al., 2017). Upon diagnosing delirium in hospitalized COVID-19 patients, attempts should be made to identify and address any underlying precipitating factors (e.g., dehydration, untreated pain, alcohol or benzodiazepine withdrawal, renal dysfunction with delayed medication clearance, etc.) (Marra et al., 2017; Oh et al., 2017). To date, no pharmacological agent has received FDA approval for the prevention or treatment of delirium. However, typical and atypical antipsychotics, benzodiazepines, propofol, and dexmedetomidine, among others, are frequently used to treat hyperactive delirium (Marra et al., 2017; Oh et al., 2017; Kotfis et al., 2020). Anecdotal observations have suggested association of COVID-19 delirium with myoclonus, rigidity, alogia, and abulia, suggesting a dopamine-depletion state or catatonia-spectrum condition, hence pragmatic, individualized step-wise treatment approach is prudent (Baller et al., 2020).

Acute stroke is another commonly reported neurologic dysfunction of COVID-19. The American Heart Association released emergency guidance at the beginning of the pandemic that recommended adherence to standard treatment guidelines whenever possible (On Behalf of the AHA/ASA Stroke Council Leadership, 2020). All patients with ischemic stroke should continue to be evaluated for potential treatment with standard-dose thrombolytics or endovascular thrombectomy (Dafer et al., 2020). While older COVID-19 patients with stroke have worse outcomes than younger cohorts, there is no evidence to suggest that it is due to a difference in therapeutic response. Given concerns for enhanced inflammation and hypercoagulability with COVID-19, there has been significant interest in using empiric therapeutic anticoagulation in place of standard chemoprophylaxis. However, trials have shown futility or even harm with empiric anticoagulation in COVID-19 patients (Lynn et al., 2021). Adhering to pre-pandemic treatment protocols is the practice standard in the management of stroke.

Patients on immunosuppressants and those with neuroinflammatory diseases like multiple sclerosis should continue treatment of their underlying illness while applying precautions to prevent viral spread and ensuring access to healthcare via teleneurology (Josephson and Kamel, 2020).

## Discussion

Elderly patients with COVID-19 are susceptible to neurologic conditions like acute stroke, acute encephalopathy, neuropsychiatric manifestations, and complications related to underlying CND. Reports from heavily affected countries like China, Italy, and the U.S. have informed that elderly population suffer a high rate of COVID-19-associated mortality (Centers for Disease Control and Prevention (CDC), 2020; Marcon et al., 2020; Wu and McGoogan, 2020). As neurologic dysfunctions of COVID-19 lead to increased morbidity and mortality, systematic studies on acute and long-term implications of neurologic complications of COVID-19 are imperative.

One of the biggest concerns of the COVID-19 pandemic is the tendency to periodically overwhelm hospitals and medical centers at the local, regional, and national level. A finite supply of healthcare resources led healthcare leaders to craft directives that address scarce resource allocation (Farrell et al., 2020a). The Italian Society of Anesthesiology, Analgesia, Resuscitation, and Intensive Care (SIAARTI) published guidelines that informed care during the outbreak in Northern Italy in March-2020 (Cesari and Proietti, 2020; Farrell et al., 2020a). Although this policy was criticized for overreliance on chronologic age and resource prioritization for younger patients, it prompted a search for more equitable criterias (Cesari and Proietti, 2020). Numerous national and international societies have released policy recommendations to guide resource allocation which involves a few common themes. First, it is inappropriate to use chronological age alone as an exclusion criteria (Farrell et al., 2020a; Montero-Odasso et al., 2020). Second, given the heterogeneity in the baseline health status of aged adults, use of objective measures such as the Clinical Frailty Scale or Sequential Organ Failure Assessment are thought to be valid and equitable alternatives in assessing potential benefit of therapeutic intervention (De Smet et al., 2020; Farrell et al., 2020b; Montero-Odasso et al., 2020). Third, implementation of protocols to prioritize advanced care planning is not only an integral component of patient-centered care but may also help in allocation of limited resources by promptly identifying patients who opt not to be intubated or resuscitated (Farrell et al., 2020a,b; Montero-Odasso et al., 2020). In one study, only 2.9% of COVID-19 patients older than 80 years survived to hospital discharge after receiving cardiopulmonary resuscitation (Hayek et al., 2020). Hence early discussions regarding goals of care is important, particularly in patients with neurologic dysfunctions. Depending on the type and severity of the disease, neurologic conditions often hold a grave prognosis, reduce life expectancy, or are associated with difficult to control pain and depression (Boersma et al., 2014; Creutzfeldt et al., 2018). Additionally, caregivers of patients with neurologic conditions are known to have similar rates of distress and burnout as that of cancer patient caregivers (Kim and Schulz, 2008). In severely ill patients with persistent encephalopathy or coma requiring full time care, burden of disease is tremendous from clinical, social, and economic standpoint. Given the high burden of disease both on patients and caregivers, early goals of care discussions become extremely important in patients with neurologic dysfunction. Lastly, healthcare systems need to work on facilitating access to in patient and outpatient palliative care and hospice services for COVID-19 patients.

Strained healthcare systems and rising healthcare costs are global problems predating the coronavirus pandemic. With the additional economic burden related to severe COVID-19 illness and the short and long-term disability associated with neurologic complications, the financial hit is likely to be staggering. For perspective, the estimated annual direct and indirect costs for ~795,000 strokes in the U.S. from 2014 to 2015 was $45.5 billion, while healthcare cost related to delirium alone was $164 billion in 2011 (Inouye et al., 2014; Virani et al., 2020). Although the actual economic impact of COVID-19 and related neurologic complications is yet to be determined, it is likely to have profound financial implications for an extended period of time.

The scientific community is only beginning to evaluate the long-term effects of the pandemic. Robust long-term data are lacking, especially pertaining to neurologic complications. One of the first studies to focus on outcomes after hospitalization for COVID-19 found that 44% of Italian patients rated their quality of life as worse (≥10 point difference on a scale of 100) since contracting COVID-19. Though patients were assessed at a mean of 60.3 days since symptom onset, 87.4% reported at least one ongoing COVID-19-related symptom, particularly dyspnea and fatigue (Carfì et al., 2020). Prolonged symptoms are also reported among those with mild COVID-19 infections. In a telephone survey of American adults with mild COVID-19 illness conducted 14–21 days after a positive PCR test, 47% of respondents aged ≥50 years reported ongoing symptoms (Tenforde et al., 2020). Data on COVID-19-associated neurologic conditions including inflammatory, vascular, autoimmune, and neurodegenerative diseases are urgently needed (Wang et al., 2020). Similarly, neuropsychiatric conditions related to social isolation are only just beginning to surface and are likely to have significant long-term implications.

In conclusion, COVID-19 has been a catastrophic pandemic, particularly for the elderly, who tend to suffer from neurologic complications as well as a very high rate of morbidity and mortality. The physical, emotional, psychological, and financial implications of this disease have been severe. Long-term data are still needed to understand the lasting complications of this devastating pandemic.

## Author Contributions

SM: substantial contributions including conception and design of the work, literature review, interpretation and summarization of data, drafting the complete manuscript, revising it critically for important intellectual content, and final approval of the manuscript to be published. MD: contribution including conception and design of the work, literature review, interpretation and summarization of the data, drafting of critical portion of the manuscript, critical revision for important intellectual content, and final approval of the manuscript. All authors contributed to the article and approved the submitted version.

## Conflict of Interest

The authors declare that the research was conducted in the absence of any commercial or financial relationships that could be construed as a potential conflict of interest.
